# COVID-19: Soziale Arbeit auf der Suche nach ihrem Auftrag im Katastrophenfall

**DOI:** 10.1007/s12054-020-00284-5

**Published:** 2020-04-01

**Authors:** Caroline Schmitt

**Affiliations:** Mainz, Deutschland

**Keywords:** COVID-19, Corona-Pandemie, Katastrophe, Katastrophenhilfe, Solidarität, Vulnerabilität, Soziale Arbeit

## Abstract

COVID-19 stellt die Soziale Arbeit vor neue und alte Herausforderungen. Die Pandemie macht den Ausschluss marginalisierter Personengruppen und globale Ungleichheiten besonders sichtbar. Der Beitrag versteht die Corona-Pandemie als „Katastrophe“ und stellt erste Überlegungen dazu an, wie Soziale Arbeit ihren gesellschaftlichen Auftrag angesichts von COVID-19 ausgestalten und sich nachhaltig in der Katastrophenhilfe verankern kann.

COVID-19 macht gesellschaftliche Problemlagen und globale Ungleichheiten besonders deutlich – wie etwa Missstände im Gesundheitswesen und in Altenpflegeheimen oder die Segregation geflüchteter Menschen in Großunterkünften und Lagern mit unzureichenden hygienischen Bedingungen. Zugleich verhalten sich Menschen in dieser schwierigen Zeit solidarisch und unterstützen einander in ihren Nachbarschaften, den Städten und in digitalen Medien. Welche Herausforderungen und Potentiale zeigen sich angesichts des „Katastrophenfalls COVID-19“ für eine Soziale Arbeit, die niemanden vergessen will?

SARS-CoV‑2 – kurz COVID-19 oder Coronavirus genannt – bestimmt gegenwärtig unserer aller Alltag. Ende des Jahres 2019 nahmen noch längst nicht alle von der Meldung dieses neuen Virus Notiz, das sich mittlerweile global ausgebreitet hat und an nationalstaatlichen Grenzen, unterschiedlichen Lebensaltern und prekären gesellschaftlichen Lebenslagen keinen Halt macht. COVID-19 entfaltet Wirkmacht und macht theoretische Diskussionen zu den globalen Auswirkungen von zunächst lokal erscheinenden Ereignissen für uns alle ganz unmittelbar erfahrbar. Dieser Beitrag zeigt in einem ersten Schritt, wie Menschen mit der Corona-Pandemie umgehen und stellt Solidaritäten wie Leerstellen von Hilfe gleichermaßen heraus. In einem zweiten Schritt verortet er die Corona-Pandemie als globale und gesellschaftliche Katastrophe und entfaltet vor diesem Hintergrund die Aufgabe, Soziale Arbeit als Katastrophenhilfe zu konzeptualisieren.

## Solidarität in Krisenzeiten

In den Medien häufen sich die Meldungen zu solidarischer Unterstützung in einer gesellschaftlichen Problemlage, die im öffentlichen Diskurs mit dem Schlagwort „Corona-Krise“ bezeichnet wird. Eine solidarische Verbundenheit der Bevölkerung zeigt sich in dieser Krise an vielen Beispielen. Ähnlich wie in Spanien und Italien applaudierten auch in Deutschland am 18. März 2020 vielerorts Menschen auf ihren Balkonen und an ihren Fenstern. Sie drücken ihren Dank an Ärzt_innen, Mitarbeitende im Einzelhandel und das Pflegepersonal in Heimen aus, die eine medizinische Versorgung und den Verkauf von Lebensmitteln am Laufen halten. Nachbarschaftshilfen organisieren Hundeausgeh-Services und Einkäufe für lebensältere Menschen (MDR 2020) und in den sozialen Medien teilen Menschen die Aufforderung, zu Hause zu bleiben, um die Ansteckung durch das Virus für alle einzudämmen. In Berlin hat der Schüler Noah Adler das Bürger_innenportal „Coronaport“ (https://www.coronaport.net/) ins Leben gerufen. Die Plattform versteht sich als Schnittstelle. Sie koordiniert Angebot und Bedarf an Unterstützung und bringt Helfer_innen mit Menschen mit Unterstützungsbedarf zusammen. In den Supermärkten werden mancherorts Einkaufszeiten für lebensältere Menschen eingerichtet, damit sie möglichst wenig in Kontakt mit anderen kommen. Auch die Bundeskanzlerin Angela Merkel appellierte in ihrer Fernsehansprache an die Solidarität der Menschen, sich rücksichtsvoll zu verhalten und im Sinne aller die sozialen Kontakte einzuschränken (Braun 2020). Was drücken diese exemplarischen Beispiele solidarischen Handelns aus? Der Soziologe Heinz Bude formuliert dies in einem Interview mit Christian Bangel folgendermaßen: COVID-19 schaffe eine kollektive Erfahrung von Verwundbarkeit. Hieraus ergebe sich „ein Gefühl wechselseitiger Sorge“ und „Verantwortung“ (Bangel 2020, o.S.), das uns noch länger beschäftigen werde.

## COVID-19 als soziale Krise

Zeitgleich zu Erfahrungen von Solidarität macht die Corona-Krise alte und neue Exklusionsmechanismen deutlich: Für die Menschen in Geflüchtetenlagern – ob auf der Insel Lesbos oder in den großen Unterkünften in Deutschland – ist bisher keine systematische Änderung der Lebensumstände in Sicht. In Lagern und Großunterkünften ist eine physische Distanzhaltung aufgrund der räumlichen Enge nicht oder kaum umsetzbar. Die hygienischen Umstände sind schwierig. In improvisierten Camps weltweit sind häufig keine sanitären Anlagen vorhanden. Auch in Wohngegenden, die im öffentlichen Diskurs als „Slums“ bezeichnet sind, stellen die Enge des Zusammenlebens und -wohnens und ein häufig nur unzureichender Zugang zu sauberem Wasser und Seife ein großes Risiko im sozialen Miteinander dar. In Krankenhäusern und Altenpflegeheimen sollen Besuchsverbote lebensältere Menschen vor einer Infektion mit dem Virus schützen. Aufgrund von Personalknappheit und der Ökonomisierung des Gesundheitswesens drohen soziale Sorge und Kommunikation in einer besonders schweren Zeit für die betreffenden Menschen noch weiter zu schwinden. Von Armut bedrohte Menschen sehen sich mit der Verschärfung eines ohnehin schon herausfordernden Lebensalltags konfrontiert: Die „Tafeln“ haben zu Teilen in Deutschland geschlossen. Die Versorgung mit Lebensmitteln ist mehrheitlich auf einen Konsum der benötigten Produkte in den noch geöffneten Supermärkten beschränkt.

Was bedeutet eine längerfristige Auseinandersetzung mit der Corona-Pandemie, wie sie Bude in dieser Gemengelage von Solidarität und Benachteiligung fordert, für die Soziale Arbeit?

## Herausforderungen und Potentiale für die Soziale Arbeit

Die angeführten Beispiele machen eines deutlich: Die Ausbreitung des Virus stellt Vulnerabilitäten von Menschen in schwierigen Lebenssituationen und ausschließende gesellschaftliche Strukturen besonders deutlich heraus – und verschärft sie noch. Welche Herausforderungen gilt es angesichts sich verschärfender Exklusionen aufzugreifen? Welche Potentiale kann die Soziale Arbeit hierbei nutzen? Und welche Lobby hat sie in diesen schwierigen Zeiten?

COVID-19 fungiert für die Soziale Arbeit – so die These des Beitrags – als reflexive Vergegenwärtigung unserer gesellschaftlichen Lage, der wir uns aus der Krise heraus (neu) nähern können. Die Soziale Arbeit darf angesichts der Gefahr, dass sich die Lebenssituationen aller, jedoch mancher im Besonderen, durch die Krise verschärfen, nicht schweigen.

## COVID-19 als „Katastrophe“

Was kann Soziale Arbeit also tun? Hierzu kann ein globaler Blick hilfreich und wegweisend sein und ein Verständnis von COVID-19 nicht nur als gesundheitliche und wirtschaftliche, sondern als grundlegend gesellschaftliche „Katastrophe“. Zum Umgang mit Katastrophen haben die Vereinten Nationen im Jahr 2000 das „United Nations Office for Disaster Risk Reduction“ (UNDRR) eingerichtet. Es wurde geschaffen, um das damals auf den Weg gebrachte internationale Rahmenprogramm „United Nations International Strategy for Disaster Reduction (UNISDR)“ zur Reduzierung von Katastrophenrisiken umzusetzen (BMZ 2015, S. 19). Seit 2015 steht die Umsetzung des „Sendai Framework for Disaster Risk Reduction 2015–2030“ im Fokus. Ziel dieses Rahmenwerks ist die globale Reduzierung und Prävention von Katastrophen. Eine Katastrophe wird in diesem Zusammenhang wie folgt definiert:

A serious disruption of the functioning of a community or a society causing widespread human, material, economic or environmental losses which exceed the ability of the affected community or society to cope using its own resources. A disaster is a function of the risk process. It results from the combination of hazards, conditions of vulnerability and insufficient capacity or measures to reduce the potential negative consequences of risk (UNISDR 2004, S. 1).

Eine Katastrophe meint in diesem Verständnis ein Ereignis, das Communities maßgeblich in ihren Routinen tangiert und weitreichende Auswirkungen auf das soziale Zusammenleben und die ökonomischen und materiellen Ressourcen von Gesellschaften hat. Reichen die üblichen Strukturen von Gesellschaften nicht mehr aus, die Verluste aufzufangen, die mit Katastrophen wie Pandemien oder etwa Naturkatastrophen und Kriegen einhergehen, erhöhen sich die Vulnerabilitäten der Menschen. Je ohnmächtiger diese der Katastrophe gegenüberstehen, desto vulnerabler sind sie. COVID-19 lässt sich nach dieser Definition durchaus als globale Katastrophe verstehen, die nach neuen gesellschaftlichen Wegen verlangt, um Handlungsfähigkeit auf globaler, nationalstaatlicher, Community- und individueller Ebene (wieder) herzustellen.

## Soziale Umwelten nachhaltig gestalten

Dieses umfassende Verständnis von Katastrophen korrespondiert mit breit angelegten Strategien zu ihrer Reduktion. UNISDR und UNDRR sprechen in diesem Zusammenhang von Katastrophenrisikoreduktion. Hiermit ist eine grundlegende Gestaltung nachhaltiger Umwelten gemeint, um die Handlungsfähigkeit von Menschen im Katastrophenfall zu erhöhen (ebd.). Einzelne, Communities, Länder, Nationalstaaten, globale Lebensumwelten und gesundheitliche, kulturelle, sozioökonomische und ökologische Fragen sollen gleichermaßen in den Blick geraten: Die Resilienz dieser Umwelten zu stärken, ist umfassendes Ziel (UNDRR 2015, S. 4). Das Sendai Framework wurde von der Europäischen Union und Deutschland unterzeichnet. EU wie Deutschland haben sich zur Umsetzung einer solchen Katastrophenrisikoreduktion auf Regierungs- und EU-Ebene verständigt. Genau an dieser Stelle zeigen sich Anschlusspunkte auch für die Soziale Arbeit.

## Soziale Arbeit im Katastrophenfall

Das Verständnis von Katastrophenrisikoreduktion des UNDRR korrespondiert mit dem Verständnis einer Sozialen Arbeit, die sich als zuständig für Communities, soziale Entwicklung und Inklusion begreift (IFSW 2014). Die Corona-Pandemie kann aus diesem Blickwinkel heraus ein Anstoß dazu sein, Soziale Arbeit als Katastrophenhilfe weltweit und in Deutschland zu konzeptualisieren, sich dabei in internationale Netzwerke und Diskussionen zu involvieren und bisherige Arbeiten in diesem Themenfeld zu würdigen und aufzugreifen. So hat Treptow (2007) in einem Sammelband das Verhältnis von Katastrophenhilfe und humanitärer Hilfe bereits vor mehr als einem Jahrzehnt reflektiert und damit ein Feld Sozialer Arbeit herausgestellt, das aus aktuellem Anlass (wieder) ganz zentral auf die Bühne der Auseinandersetzung rückt. Auch Bähr (2011, 2014) hat die Notwendigkeit Sozialer Arbeit als Katastrophenhilfe thematisiert – dies u. a. vor dem Hintergrund der Anschläge vom 11. September 2001. Die Corona-Pandemie ist nun erneutes Szenario, um Soziale Arbeit als Katastrophenhilfe zu denken und ein solches Verständnis grundlegend in der Sozialen Arbeit zu verankern.

## Eckpfeiler Sozialer Arbeit als Katastrophenhilfe

Wie kann eine solche Verankerung aussehen? Die Wellen an Solidarität, die sich in diesen Tagen zeigen, können hierbei zum einen Inspiration, zum anderen Gegenstandsfeld einer Sozialen Arbeit als Katastrophenhilfe sein. Soziale Arbeit kann dabei unterstützen, Infrastrukturen solidarischer Hilfe mit zu gestalten, wenn sie an dieser Stelle gebraucht wird. Solidarische Formate wie die Onlineplattform „Coronaport“ können gar als Vorbild dienen, um auch Angebote Sozialer Arbeit in Zeiten, in denen Menschen ihr soziales Leben vor der eigenen Haustüre einschränken müssen, auf digitalem Wege verfügbar zu machen. Menschen in besonders großer Sorge, die etwa unter einer Angststörung leiden, Menschen, die in Pflegeheimen oder in der „Solo-Quarantäne“ zu vereinsamen drohen sowie Menschen ohne Versorgung mit Medikamenten und ohne Zugang zu sauberem Wasser und Seife werden in Zeiten der Krise besonders vulnerabel gemacht.

Diese ausgrenzenden Lebenslagen gilt es im Fall von Katastrophen (und darüber hinaus) ganz zentral auf die Agenda zu setzen und an einer grundlegenden Verbesserung dieser Lebenslagen gemeinsam mit den Betreffenden auch jenseits der Katastrophe zu arbeiten. Gestärkte Communities – so lässt sich folgern – können in Krisenzeiten besser auf eine Pandemie und andere Katastrophen wie Naturkatastrophen reagieren. Vor dem Hintergrund von Leerstellen der Versorgung ist die Corona-Pandemie Anlass genug, darüber nachzudenken, wie eine mobile Soziale Arbeit Menschen in Notlagen gut erreichen kann, ob virtuell oder konkret physisch. Eine Weiterentwicklung digitaler Formate translokalen Sorgens ist auch „jenseits der Krise“ von hoher Relevanz.

## Überlegungen der International Federation of Social Workers

Diese Überlegungen fügen sich in eine erste Systematisierung zu Aufgaben Sozialer Arbeit in der Corona-Pandemie ein, wie sie die International Federation of Social Workers (IFSW) am 16. März 2020 vorgelegt hat (IFSW 2020):

- Soziale Arbeit solle angesichts des Virus nicht in Angst verharren;
- Soziale Arbeit solle sich gegen Falschmeldungen einsetzen, die Angst verbreiten und die Menschen verunsichern;
- Soziale Arbeit solle den Strategien physischer Distanzhaltung und Hygienemaßnahmen entsprechen;
- Soziale Arbeit solle gemeinsam mit anderen Fachkräften auf die Sorgen der Menschen eingehen;
- Soziale Arbeit solle eine Aufgabe in „disaster response for the vulnerable (elderly, breast feeding/pregnant mothers, people with disability, and children)“ (ebd., o.S.) übernehmen und die Deckung menschlicher Grundbedürfnisse organisieren;
- Soziale Arbeit solle sich in Nachbarschaftshilfen involvieren und Sicherheitsvorkehrungen mit organisieren;
- Soziale Arbeit solle strategische Kommunikation online und über Telefon und Ansprechpartner_innen für Menschen mit Unterstützungsbedarf bereitstellen.

## Nachhaltige Infrastrukturen der Unterstützung

Die aufzeigten Überlegungen verdeutlichen, dass Katastrophen wie die Corona-Pandemie ganz zentral die Lebenswelten von Adressat_innen sowie Handlungs- und Arbeitsfelder Sozialer Arbeit tangieren. Katastrophen sind Ereignisse, die zwingend mit einer Reaktionsweise Sozialer Arbeit einhergehen müssen. Die Corona-Pandemie lässt sich in diesem Sinne als Möglichkeit und Chance begreifen, eine Konzeptualisierung Sozialer Arbeit als Katastrophenhilfe weiter voranzutreiben. Hierbei kann die Soziale Arbeit von zivilgesellschaftlichen Unterstützungsformaten, die flexibel und zeitnah auf die Corona-Pandemie reagieren, lernen.

Gewährung von und Zugang zu Unterstützung darf im Katastrophenfall jedoch nicht dem Zufall überlassen und einseitig in die Hände von Freiwilligen gelegt werden – die unbestreitbar bedeutsame Formen von Unterstützung bereitstellen. Qua ihres gesellschaftlichen Mandats muss sich Soziale Arbeit auch und insbesondere *im Katastrophenfall als zuständige professionelle Instanz begreifen*, da Katastrophen marginalisierte Personengruppen weiter an den gesellschaftlichen Rand zu drängen drohen und für alle Menschen mit hohen Belastungen einhergehen. Soziale Arbeit versteht sich in einem solchen Verständnis als Instanz, die sich Katastrophen aus einer professionellen Perspektive heraus annimmt und *Unterstützung vor dem Hintergrund des Mandats einer Menschenrechtsprofession* (Staub-Bernasconi 2007) koordiniert. Sie hat dann den Auftrag, den *Zugang zu sozialen Dienstleistungen sicherzustellen, Exklusionsmechanismen im Katastrophenfall zu vermeiden* und Folgen und Risiken von Katastrophen für alle Menschen zu bedenken und hierbei besonders vulnerabilisierte Gruppen in die Debatte einzubinden.

Diese Aufgabe geht über die Bearbeitung eines einzelnen Katastrophenfalls hinaus. Denn: Gelingende Infrastrukturen der Unterstützung vermögen insbesondere in Zeiten der Krise Risiken und Gefährdungen zumindest zu puffern. Ein *Mitdenken von Katastrophen zeigt sich als Querschnittsaufgabe in allen Bereichen Sozialer Arbeit*, wenngleich explizite Herausforderungen im konkreten Fall von Katastrophen denkbar an spezifische Instanzen Sozialer Arbeit zu koordinieren sein könnten: Ist ein *„Katastrophen-Team Sozialer Arbeit“*, das sich etwa auf die Versorgung allein lebender Menschen und Menschen in Heimkontexten fokussiert, für einen Krisenfall vorausschauend planbar, sodass im Fall von Pandemien, Naturkatastrophen oder Anschlägen schnell agiert werden kann? Braucht es zusätzlich zu einer grundlegenden Sensibilisierung für Katastrophen als Querschnittsthema eigene Instanzen Sozialer Arbeit, welche *die sozialen Dienste auf ihre „Reaktionsfähigkeit im Katastrophenfall“ hin abklopfen*, diese Infrastrukturen mit allen Beteiligten gemeinsam erweitern und im Katastrophenfall koordinierend aktiv werden?

Mit diesen Fragen gehen weitere Punkte einher – wie Fragen der Finanzierung eines *sozialarbeiterischen Katastrophenmanagements* sowie Fragen der *Qualifikation von Fachkräften*. Denkbar ist eine *Involvierung Sozialer Arbeit als wichtige Schaltstelle in die Bestrebungen zur Katastrophenrisikoreduktion*, wie sie im Rahmen des Sendai Rahmenpapiers auch in Deutschland, aber auch in anderen Ländern vorangetrieben werden soll. Zudem ist Katastrophenhilfe eine Ländergrenzen überschreitende und *interprofessionelle Aufgabe*. Die IFSW könnte hierbei Bindeglied einer sich international ausrichtenden Sozialen Arbeit als Katastrophenhilfe sein.

Interprofessionelle Netzwerke sind im und jenseits des Katastrophenfalls für die Soziale Arbeit zentral. Die Corona-Pandemie kann zu einem erhöhten Bedarf psychologischer Betreuung, einem erhöhten Bedarf an Schuldner_innenberatung oder auch lebenspraktischer Alltagshilfe und einem erhöhten Bedarf digital zur Verfügung stehender Informationen führen und damit die Medienpädagogik besonders herausfordern – um nur einige Beispiele zu nennen.

Diese Überlegungen gilt es angesichts der Corona-Pandemie grundlegend weiterführend zu diskutieren. Bedeutsam erscheint die Zieldimension, *unterstützende Infrastrukturen nachhaltig zu gestalten* sowie ein Verständnis von Katastrophenhilfe zu entwickeln, das über ein unmittelbares Reagieren auf Katastrophen hinausreicht und die sozialen Folgen von Katastrophen durch die *Gestaltung resilienter Umwelten und durchdachter Hilfeangebote bereits vor Eintritt von Katastrophen *thematisch macht. Nachhaltig, interprofessionell, aber auch spezifisch im Anliegen, niemanden in Katastrophen zu vergessen, so ließe sich schlussfolgern, könnten Perspektiven einer Sozialen Arbeit als Katastrophenhilfe sein. COVID-19 fungiert damit als Warnung und Wegweiser zugleich, Soziale Arbeit in einer globalen Welt entsprechend auszurichten.
